# Non-Contact and Self-Calibrated Photopyroelectric Method for Complete Thermal Characterization of Porous Materials

**DOI:** 10.3390/ma16155242

**Published:** 2023-07-26

**Authors:** Mohanachandran Nair Sindhu Swapna, Carmen Tripon, Robert Gutt, Alexandra Farcas, Marcel Bojan, Dorota Korte, Irina Kacso, Mladen Franko, Dorin Dadarlat

**Affiliations:** 1 Laboratory for Environmental and Life Sciences, University of Nova Gorica, Vipavska 13, SI-5000 Nova Gorica, Slovenia; swapna.nair@ung.si (M.N.S.S.); dorota.korte@ung.si (D.K.); mladen.franko@ung.si (M.F.); 2 National R&D Institute for Isotopic and Molecular Technologies, Donat 67-103, 400293 Cluj-Napoca, Romania; robert.gutt@itim-cj.ro (R.G.); alexandra.farcas@itim-cj.ro (A.F.); marcel.bojan@itim-cj.ro (M.B.); irina.kacso@itim-cj.ro (I.K.); ddadarlat@gmail.com (D.D.)

**Keywords:** photopyroelectric calorimetry, thermal parameters, porous materials

## Abstract

A general theory of a photopyroelectric (PPE) configuration, based on an opaque sample and transparent pyroelectric sensor, backing and coupling fluids is developed. A combined back-front detection investigation, based on a frequency scan of the phase of the PPE signals, followed by a self-normalization of the phases’ behavior, leads to the possibility of simultaneously measuring both thermal effusivity and diffusivity of a solid sample. A particular case of this configuration, with no coupling fluid at the sample/backing interface and air instead of coupling fluid at the sample/sensor interface (non-contact method) is suitable for simultaneous measurement ofboth thermal diffusivity and effusivity (in fact complete thermal characterization) of porous solids. Compared with the already proposed configurations for investigations of porous materials, this novel configuration makes use of a fitting procedure with only one fitting parameter, in order to guarantee the uniqueness of the solution. The porous solids belong to a class of materials which are by far not easy to be investigated using PPE. To the best of our knowledge, porous materials represent the only type of compounds, belonging to condensed matter, which were not taken into consideration (until recently) as potential samples for PPE calorimetric investigations. Consequently, the method proposed in this paper complete the area of applications of the PPE method. Applications on some porous building materials and cellulose-based samples validate the theory.

## 1. Introduction

During the last decades, the photothermal methods (PT) attracted an increased attention as tools for the direct measurement of the static and dynamic thermal parameters of condensed matter samples. Amongst the PT methods, the photopyroelectric (PPE) technique proved to be one of the most performant. In a PPE calorimetry, a laser radiation is absorbed into the sample, and consequently is generating heat. Afterwards, this heat is measured with a pyroelectric sensor which is located in thermal contact with the sample [1,2,3]. One or two thermal parameters can be directly measured by using the PPE method in various detection configurations [4,5]. It is well known that the static thermal parameter, the volume specific heat, C, is linked with the three dynamic thermal parameters, thermal conductivity, *k,* diffusivity, *α,* and effusivity*, e*, by two relationships [4,5]and consequently, if you want a complete thermal characterization of a material, you need to directly measure at least two of them.

Concerning the type of samples suitable to be investigated by PPE, the best are liquids, because the thermal contact between a liquid sample and a solid sensor is perfect [5]. When investigating solid compounds, a coupling fluid is placed between the sensor and sample, to assure a good thermal contact [4,6,7,8]. However, a liquid or paste coupling fluid can be used only for non-porous solid samples, because in the case of porous materials, such a coupling fluid will penetrate the sample, what causes false results. The only option to thermally investigate such porous samples, with the PPE method, is to replace the liquid/pasty coupling fluid between the sensor and sample with a layer of air.

Salazar et al. [9] proposed for the first time an alternative configuration that uses air as a coupling fluid between the pyroelectric sensor and a solid sample, with application to bad thermal conductors. In this method, the detection is performed using an opaque pyroelectric sensor, which leads to the direct measurement of the sample’s thermal effusivity by performing a fit of the experimental data with three fitting parameters: sample’s thermal effusivity, heat losses by convection and radiation at the irradiated surface and air gap between sensor and sample. There are two main limitations of this approach: (i) concerning the thermal parameters affecting the output of the experiment, only the thermal effusivity can be measured; (ii) concerning the fitting procedure, the multiparametric fit with three fitting parameters can lead sometimes to degenerate solutions. The method proposed in Ref. [9] has been already improved in two steps. The first step was more or less formal and consisted in the limitation of the range of variation of two of the fitting parameters [10] (the heat losses by convection and radiation and air gap between sensor and sample) to limit the degeneracy of the results. In the second step, the convection of the air located in front of the irradiated electrode of the sensor was removed, through a new detection configuration; in such a way the final fit was based only on two fitting parameters [11]. However, even if the methods proposed in Refs. [10,11] are improvements concerning the possible degeneracy of the results, a limitation persists: only the thermal effusivity can be directly measured by the proposed configurations.

It seems that a method able to measure simultaneously both thermal diffusivity and effusivity of a porous solid and which makes use of a fitting procedure with only one fitting parameter (in order to guarantee the uniqueness of the solution) is a request. This is the key motivation for the performed research.

For this purpose, in this paper, we try to generalize and adapt for the investigation of porous solids, a method, proposed initially by Zammit et al. [12]. The method is based on two consecutive measurements (frequency scans of the PPE signal), one in back PPE configuration and one in front PPE configuration, respectively, followed by a self-normalization of the phase vs. modulation frequency behaviors. This technique uses transparent pyroelectric sensors and backing materials, and therefore the self-normalized PPE phase depends only on the sample’s thermal effusivity and diffusivity and on the thermal effusivity of the backing (the phase is independent of the thermal and geometrical parameters of the sensor and coupling fluid between sensor and sample). The method allows the direct measurement of the sample’s thermal effusivity in the low frequency regime given that the thermal diffusivity has been already measured in the region of high frequencies. Unfortunately, in the form presented in Ref. [12], the method proposed by Zammit et al., cannot be applied for characterization of porous solids, because: (i) the authors used a liquid coupling fluid between the sample and sensor and (ii) they also presumed a perfect thermal contact between the sample and the translucent backing material; they used a solid backing when measuring liquid samples and liquid backing when investigating solid samples. This experimental setup is unlikely when investigating porous solids (because a liquid backing will infiltrate into the sample), therefore, we have to find some experimental alternative.

## 2. Theory and Mathematical Simulations

**Figure 1:**
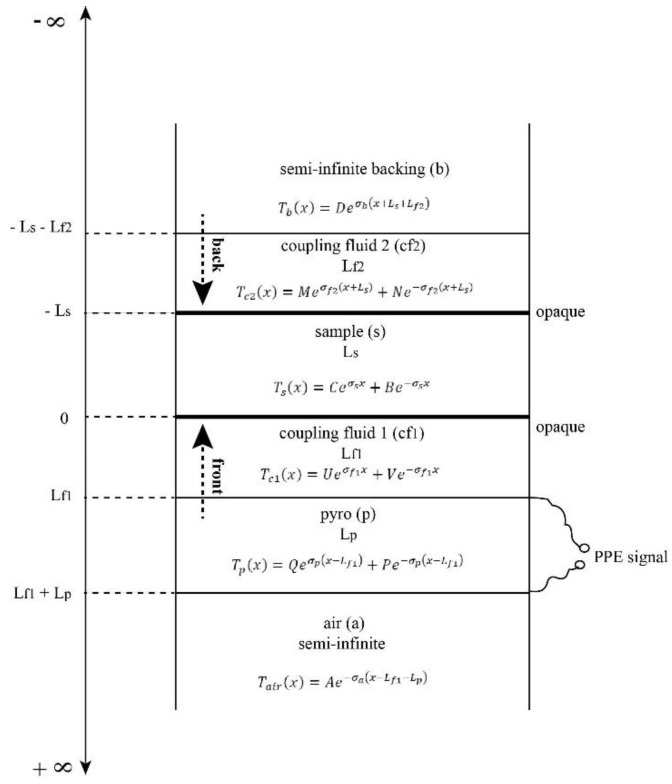
Schematic representation of the PPE detection cell used in this work.

The theory developed in the following section is applied to the layered system presented in Figure 1 with the aim to obtain a normalized PPE signal when two detection configurations “back” and “front” are considered.

The main particularity of the detection cell described above is the transparency of all layers (pyroelectric sensor, backing, coupling fluids) except the sample. As mentioned before two configurations will be considered and, in both, the heat will be generated at the sample’s opaque surfaces at *x* = 0 in front and *x* = −*L_s_* in back configuration, respectively. Figure 1 contains also the solutions for the standard one directional heat diffusion system of equations [4,9,11,12].

The steps in the development of the theory are standard (see the supplementary material for details) and we will present here only some particularities.

The temperature and flux continuity at *L_f1_* + *L_p_* and *L_f1_* interfaces lead, in both back and front configurations, to the following relationships:

$$Q=Pe^{-2\sigma_{p}L_{p}}$$

$$P+Q=P\left(1+e^{-2\sigma_{p}L_{p}}\right)$$

(1) $$Q+P=Ue^{\sigma_{f1}L_{f1}}+Ve^{-\sigma_{f1}L_{f1}}$$

$$P-Q=b_{f1p}\left(-Ue^{\sigma_{f1}L_{f1}}+Ve^{-\sigma_{f1}L_{f1}}\right)$$

After some algebra we obtain:

(2) $$U=V\frac{Z}{Y}e^{-2\sigma_{f1}L_{f1}}$$

where

(3) $$Z=\frac{1+b_{f1p}}{2}\left(1+e^{-2\sigma_{p}L_{p}}\right)-1$$

$$Y=1-\frac{1-b_{f1p}}{2}\left(1+e^{-2\sigma_{p}L_{p}}\right)$$

In fact, in order to obtain the normalized back/front PPE signal we have to calculate for each configuration the quantity $〈T_{p}\left(x\right)〉$ as follows:

(4) $$〈T_{p}\left(x\right)〉=\frac{1}{L_{p}}\int_{L_{f1}}^{L_{f1}+L_{p}}\left[Qe^{\sigma_{p}\left(x-L_{f1}\right)}+Pe^{-\sigma_{p}\left(x-L_{f1}\right)}\right]dx$$

Using Equations (1) and (2) in (4) we obtain:

(5) $$〈T_{p}\left(x\right)〉=\frac{P}{\sigma_{p}L_{p}}\left(1-e^{-2\sigma_{p}L_{p}}\right)$$

with

(6) $$P=U\cdot \frac{1-b_{f1p}}{2}\cdot e^{\sigma_{f1}L_{f1}}+V\cdot \frac{1+b_{f1p}}{2}\cdot e^{-\sigma_{f1}L_{f1}}$$

Consequently

(7) $$\frac{〈T_{p}{\left(x\right)}_{B}〉}{〈T_{p}{\left(x\right)}_{F}〉}=\frac{V_{B}}{V_{F}}$$

and the calculation of the self-normalized PPE signal reduces to the calculation of quantities *V* in back (*B*) and front (*F*) configuration, respectively (*V_B_* and *V_F_*).

In Equations (1)–(7) we used the classical notations:

(8) $$\sigma =\left(1+i\right)a=\left(1+i\right){\left(\frac{\pi f}{\alpha }\right)}^{\frac{1}{2}}$$

(9) $$\frac{k_{i}\sigma_{i}}{k_{j}\sigma_{j}}=\frac{e_{i}}{e_{j}}=b_{ij}$$

where *b_ij_* represents the effusivity ratio at the *ij* interface, *a_j_* represents the reciprocal of the thermal diffusion length in the *j* material and *f* is the modulation frequency of the incident radiation.

The main information resulted from Equation (7) is that the self-normalized signal will not depend on the thermal and geometrical properties of the pyroelectric sensor.

Using the equations for the temperature and thermal flux continuity at the remaining interfaces, we obtain for the *V* quantities the relationships:

(10) $$V_{F}=\frac{\frac{H}{2k_{s}\sigma_{s}}\left(1+\varepsilon \cdot e^{-2\sigma_{s}L_{s}}\right)}{e^{-2\sigma_{f1}L_{f1}}\frac{Z}{Y}\left[1-\frac{1+b_{f1s}}{2}\left(1+\varepsilon \cdot e^{-2\sigma_{s}L_{s}}\right)\right]+\left[1-\frac{1-b_{f1s}}{2}\left(1+\varepsilon \cdot e^{-2\sigma_{s}L_{s}}\right)\right]}$$

(11) $$V_{B}=\frac{\pi }{\frac{Z}{Y}e^{-2\sigma_{f1}L_{f1}\left[1-\frac{1+b_{f1s}}{2}\left(1+\varepsilon \cdot e^{-2\sigma_{s}L_{s}}\right)\right]+\left[1-\frac{1-b_{f1s}}{2}\left(1+\varepsilon \cdot e^{-2\sigma_{s}L_{s}}\right)\right]}}$$

where

(12) $$\varepsilon \frac{e^{-2\sigma_{f2}L_{f2}}\left(1+b_{sf2}-b_{bf2}\cdot b_{sf2}-b_{bf2}\right)-\left(1+b_{bf2}-b_{sf2}\cdot b_{bf2}-b_{sf2}\right)}{\left(1+b_{bf2}+b_{sf2}+b_{sf2}\cdot b_{bf2}\right)-e^{-2\sigma_{f2}L_{f2}}\left(1-b_{sf2}-b_{bf2}+b_{bf2}\cdot b_{sf2}\right)}$$

(13) $$\pi =\frac{H^{\prime }e^{-\sigma_{s}L_{s}}}{2k_{f2}\sigma_{f2}}\cdot \frac{\left(1-b_{bf2}\right)e^{-2\sigma_{f2}L_{f2}}+\left(1+b_{bf2}\right)}{\left(1+b_{sf2}\right)\left(1+b_{bf2}\right)-\left(1-b_{sf2}\right)\left(1-b_{bf2}\right)e^{-2\sigma_{f2}L_{f2}}}$$

and for the self-normalized PPE signal:

(14) $$U_{n}=\frac{V_{B}}{V_{F}}=\frac{H^{\prime }}{H}b_{sf2}e^{-\sigma_{s}L_{s}}\frac{\left(1-b_{bf2}\right)e^{-2\sigma_{f2}L_{f2}}+\left(1+b_{bf2}\right)}{\left(1+\varepsilon \cdot e^{-2\sigma_{s}L_{s}}\right)\left[\left(1+b_{sf2}\right)\left(1+b_{bf2}\right)-\left(1-b_{sf2}\right)\left(1-b_{bf2}\right)e^{-2\sigma_{f2}L_{f2}}\right]}$$

where *H* and *H’*are the absorbed light intensities at samples *x* = 0 and *x* = −*L_s_* interfaces.

Equation (14) indicates another advantage of this method: the normalized signal does not depend on the thermal and geometrical parameters of the coupling fluid between the sensor and sample (cf1). As a consequence, we can use at the sensor/sample interface any type of coupling fluid, including air.

Equation (14) can be used to derive the sample’s thermal effusivity and diffusivity, via a multi-parametric fit with the two above mentioned thermal parameters and coupling fluid’s thickness (at sample/backing interface) as fitting parameters. At this stage, the proposed method can be applied only to non-porous solids and it is based on a fitting procedure with three fitting parameters. The only improvement, compared with previously reported configurations [9] seems to be the fact that two fitting parameters are related to the sample (thermal diffusivity and effusivity) and only one to the experiment (sample/backing coupling fluid’s thickness).

**Figure 2:**
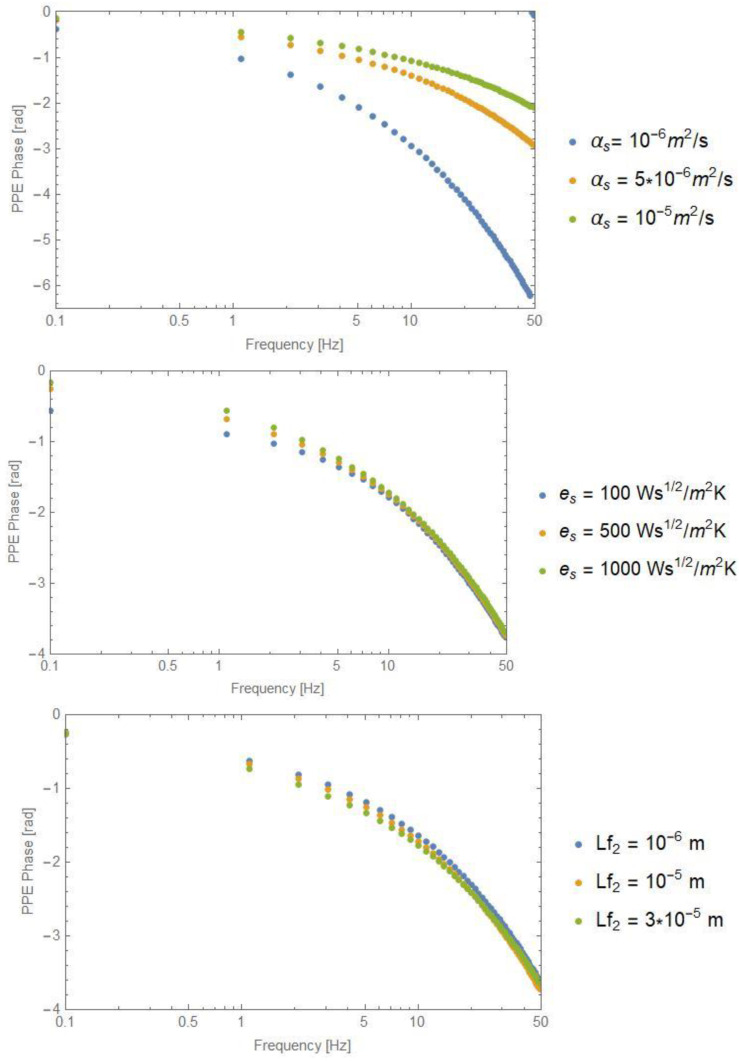
Mathematical simulations for the phase as a function of modulation frequency, as defined by Equation (14), for various values of the fitting parameters.

Some mathematical simulations of the behavior of the normalized PPE phase for different values of the fitting parameters are presented in Figure 2.

In the mathematical simulations the effusivity of the backing was considered as *e* = 800 Ws^1/2^m^−2^K^−1^, and the thermal parameters of the coupling fluid (cf2) are those for water: *e* = 1600 Ws^1/2^m^−2^K^−1^, and α_s_ = 1.45 × 10^−7^m^2^s^−1^. In the first graph of Figure 2, *e_s_* = 500 Ws^1/2^m^−2^K^−1^, and *L_s_* = 15 µm, in the second graph *L_s_* = 15 µm and *α_s_* = 3 × 10^−7^ m^2^s^−1^, in the third graph *e_s_* = 500 Ws^1/2^m^−2^K^−1^ and *α_s_* = 3 × 10^−7^ m^2^s^−1^.

Even if they describe a particular case, the mathematical simulations point out a useful feature of the configuration. At low frequencies, all three fitting parameters influence the behavior of the phase of the PPE signal. However, at high frequencies a kind of saturation concerning the thermal effusivity seems to appear, and the phase behavior is influenced mainly by the thermal diffusivity. As a consequence, if we perform a frequency scan over a large range of modulation frequencies, it seems to be possible to obtain the thermal diffusivity at high frequencies and then, to retrieve the value of the thermal effusivity in the low frequency range. The thickness of the coupling fluid (cf2) influences the behavior of the phase on the whole frequency range, its elimination as a scanning parameter will significantly improve the quality of the results, especially in connection with the “uniqueness” of the results. For this purpose, some particular cases will be derived in the following.

Case a.

The backing is in intimate thermal contact with the sample (*L_f2_* = 0). In this case, assuming $b_{sf2}=b_{sb}$ and $b_{bf2}=b_{bb}=1$, or $b_{bf2}=b_{bs}$and $b_{sf2}=b_{ss}=1$, we obtain for *ε*

(15) $$\varepsilon =\frac{b_{sb}-1}{b_{sb}+1}$$

and for Equation (14) the simplified form

(16) $$U_{n}=\frac{H^{\prime }}{H}\cdot \frac{e^{-\sigma_{s}L_{s}}}{1+b_{bs}+\left(1-b_{bs}\right)e^{-2\sigma_{s}L_{s}}}$$

in agreement with the result obtained in Ref. [12].

Case b.

The coupling fluid between sample and backing is air. In this case $b_{bf2},\;\;b_{sf2}\;\gg 1$, *ε* = 1, and Equation (14) has the form:

(17) $$U_{n}=\frac{e^{-\sigma_{s}L_{s}}}{1+e^{-2\sigma_{s}L_{s}}}$$

From experimental point of view both Equations (16) and (17) seems useful. If we calculate the phase of the normalized PPE signal in these two cases, we get:

(18) $$\varphi =-a_{s}L_{s}+tan^{-1}\left(\frac{\left(1-b_{bs}\right)sin\left(2a_{s}L_{s}\right)e^{-2a_{s}L_{s}}}{\left(1+b_{bs}\right)+\left(1-b_{bs}\right)cos\left(2a_{s}L_{s}\right)e^{-2a_{s}L_{s}}}\right);\;\mathrm{case}\;a\;$$

(19) $$\varphi =-a_{s}L_{s}+tan^{-1}\left(\frac{sin\left(2a_{s}L_{s}\right)}{e^{2a_{s}L_{s}}+cos\left(2a_{s}L_{s}\right)}\right);\;\mathrm{case}\;b\;$$

As already predicted by the mathematical simulations from Figure 2, for high enough modulation frequencies, in both cases, the second term in Equations (18) and (19) vanishes and the first term leads to the calculation of the sample’s thermal diffusivity. At low frequencies, case *a* allows the measurement of the sample’s thermal effusivity through a fit with one fitting parameter (the sample’s thermal diffusivity is already known from the investigations in high frequency range). Case *b* allows only the measurement of the sample’s thermal diffusivity in both low and high frequency ranges.

If we focus on the final target of this paper, which is the complete thermal characterization of solid porous building materials, one can use case *b* for the investigation of the sample’s thermal diffusivity, together with the method proposed in Ref. [10] for thermal effusivity investigation, or he can use directly case *a* for the measurement of both dynamic thermal parameters mentioned above.

Some comments concerning the particular case *a* are necessary. From atheoretical point of view, Equation (18) is similar to the one proposed by Zammit et al. [12]. However, if we want to apply the configuration to porous solids we have to face some experimental problems. The air is a bad thermal conductor and, as a sample/sensor coupling fluid, acts as a low-pass filter in a frequency dependent experiment, especially in the back detection configuration; an adequate frequency range must be chosen. Concerning the backing material, it has to fulfill several requests. First of all, it must stick on sample’s surface without penetrating into the pores. It must be transparent in both visible (for the laser source) and the infrared (IR) range. The transparency in IR is necessary because the sample’s irradiated surface in back detection configuration generates heat (IR radiation), that cannot be absorbed by the backing; the absorption of the heat by the backing can generate a secondary heat source and consequently, the whole theory, elaborated before, becomes useless. Another important request concerning the backing material is the value of its thermal effusivity. As demonstrated in Ref. [12], the method is sensitive to the sample’s thermal effusivity only if the ratio of the two sample/backing effusivities is in the range 0.1–10.

## 3. Experimental

The experimental set-up used in this work is typical for PPE experiments [4,5]. The radiation source was a 200 mW YAG laser, modulated from its internal power supply. The pyroelectric sensor was a 10 × 10 × 0.4 mm^3^ LiTaO_3_ single crystal provided with ITO electrodes on both sides. A SR-830 lock-in was used for data acquisition and a PC with adequate software for data processing and modulation frequency control. The measurements, in both front and back configurations, were scans of the phase of the PPE signal as a function of the modulation frequency. The information is collected from the phase of the signal due to the fact that, as demonstrated before, the phase is less noisy than the amplitude of the signal. The modulation frequency range, the power and the diameter of the laser beam were properly selected to assure the approximation for one-directional propagation of the heat through the detection cell and a reasonable signal to noise (S/N) ratio. As it is well-known the lowest S/N ratio is obtained in the back detection configuration, at high frequencies; 20 was the minimum S/N accepted value. All the measurements have been performed at room temperature. Three measurement runs were performed for each sample in order to achieve the required repeatability of the measurements.

Adequate computer programs have been used for data acquisition, frequency control and fitting procedure. For parametric identification we used the Levenberg-Marquardt algorithm using the Find Fit function in the Mathematica program.

As backing material, we tested two solid gels, one was a bergal gel used in shoes industry and the second one is a polydimethylsiloxane (PDMS) gel. Both stick on the sample without penetrating the pores. However, some of the optical and thermal properties are slightly different for the two materials.

**Figure 3:**
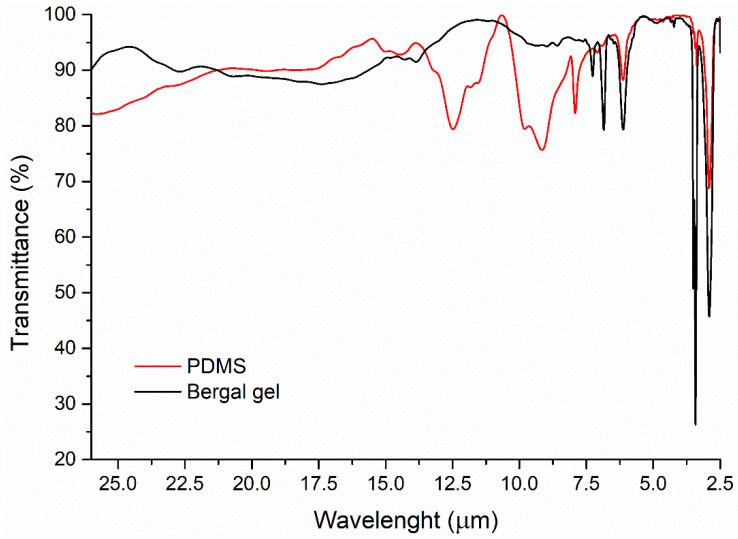
FTIR spectra for the two gels investigated as possible backing layers in the detection cell.

Figure 3 contains the IR spectra of the two gels. The FTIR spectra were recorded using a Jasco FT/IR-6100 spectrometer in the 2.5 to 26 µm spectral range, in transmittance mode with 4 cm^−1^ resolution by the KBr pellet technique. Each sample has been dispersed in about 300 mg of anhydrous KBr and mixed with an agate mortar. The pellets were obtained by pressing the mixture into an evacuated die. The spectra were collected and analyzed with Jasco Spectra Manager v.2 software.

The vibrational bands identified in the PDMS spectrum are: 13.0–8.0 µm spectral domain (δ and γCH—12.5, 11.8, 11.5, 9.8, 9.1 and 7.9 µm), 7.1 µm, 6.9sh µm (νC-C aromatic), 6.1 µm (νCOO), 3.5 and 3.4 µm (assym. and symm νCH), 2.9 µm (νOH).

The spectrum of Bergal gel presents following vibrational bands: 13.0–8.0 µm spectral domain (δ and γCH—13.8, 8.9, 8.6 and 7.2 µm), 6.8 µm (νC-C aromatic), 6.1 µm (νCOO), 3.5 and 3.4 µm (assym. and symm νCH), 2.9 µm (νOH).

Comparative analysis of the FTIR spectra of the two materials indicates that both gels have a good transparency (larger than 80% transmittance) in the whole IR range (excepting some narrow absorption bands—mentioned before—in near IR), so, both are considered suitable backings from this point of view.

**Figure 4:**
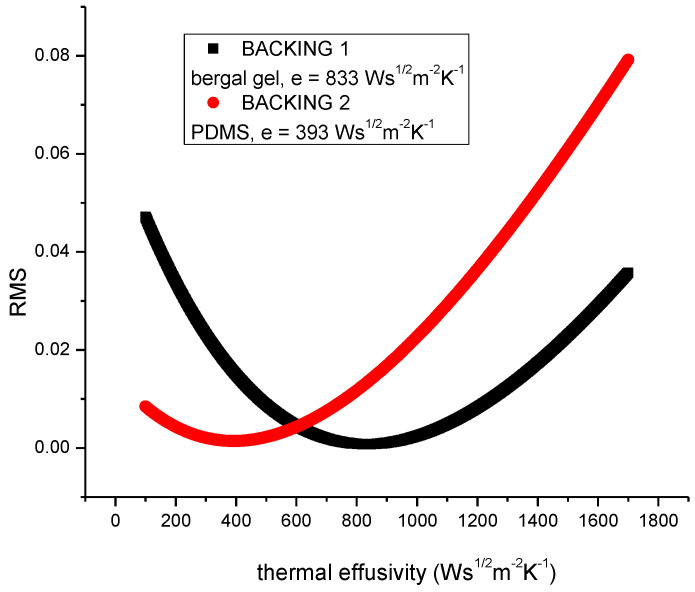
Root-mean-square as a function of sample’s thermal effusivity as obtained for the best fit performed for the normalized PPE phase. The minimum of the graph gives the value of sample’s thermal effusivity.

Concerning the value of the thermal effusivity of the backing materials, it was measured in the classical front detection configuration with the method proposed in [13]. The thermal data for the sensor were α_p_ = 1.12 × 10^−6^ m^2^s^−1^ and e_p_ = 3600 Ws^1/2^m^−2^K^−1^ [14]. Figure 4 contains the results obtained for the thermal effusivity. It presents the RMS of the fit performed in order to find the thermal effusivity. The mathematics of this calculation can be found in Ref. [13]. It is to note only that the best fit is associated with the minimum of the graphs RMS vs. thermal effusivity. Consequently, the values of the thermal effusivity for the two gels are 833 Ws^1/2^m^−2^K^−1^ for bergal gel and 393 Ws^1/2^m^−2^K^−1^ for PDMS gel. Finally, we decided to use in our experiments the PDMS due to its better adherence on the sample’s surface and due to its lower effusivity (the porous solids have generally low values for thermal effusivity).Some more details about PDMS can be found in Refs. [15,16]. It is an elastomer with excellent optical, electrical and mechanical properties, which makes it a ideal candidate for several engineering applications. The chemical formula of PDMS is CH_3_[Si(CH_3_)_2_O]_n_Si(CH_3_)_3_, where n is the number of repeating monomer [Si(CH_3_)_2_O] units. PDMS is also chemically inert, thermally stable, permeable to gases, exhibits isotropic and homogeneous properties.

Concerning the samples under investigation, some preliminary measurements have been performed on a non-porous solid, Cr_2_O_3_ single crystal. This material was largely investigated in the past, especially in connection with its antiferro-paramagnetic phase transition [17]; however, its thermal parameters around room temperature are well-known [4,12,17]. The Cr_2_O_3_ sample is a disk of about 5 mm in diameter and 500 µm thickness. In some experiments high vacuum silicone grease was used both as cf1 and cf2 coupling fluids; its thermal parameters were α = 0.1 × 10^−6^ m^2^s^−1^ and e = 730 Ws^1/2^m^−2^K^−1^ [6,7]. Other investigated samples were various types of paper (commercially available) and some porous building materials (brick, limestone, wood (fir)). Two chitosan and cellulose composite samples are prepared in the ratio 75:25 and they are enriched with 30% natural sporopollenin microcapsules (cellulose (1)) by a solution mixing procedure as described in the literature [18,19]. The sporopollenin is incorporated to improve the porosity of the chitosan-cellulose bio-composite for encapsulating the antibiotics (cellulose (2)). The two chitosan-cellulose samples have a different porosity (cellulose (1)—total ×10%—1.05, open ×10%—0.09; cellulose (2)—total ×10%—0.55, open ×10%—0.073) and consequently, different thermal parameters.

Concerning the samples preparation, all samples were prepared as disks with a thickness smaller than 1mm and flat surfaces. The surfaces have been polished with grinding papers of gradually quality, the last granulation being 1200. The quality of the samples surfaces is directly connected with the accuracy of the measurements. The most important surface of the sample is the one in contact with the backing; it must be perfect flat in order to allow for a very good adherence of the backing gel to the sample. Any imperfection can lead to incorrect results.

## 4. Results

**Figure 5:**
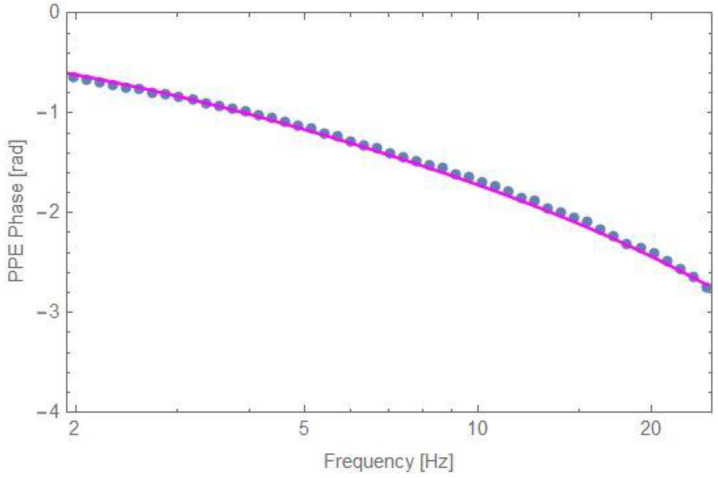
The best fit (solid line) of the experimental data (dots) for Cr_2_O_3_to Equation (14).

In order to validate Equation (14), an investigation was performed on Cr_2_O_3_ single crystal. Figure 5 presents the best fit performed with Equation (14) on the results obtained in the described configuration. The measurement was performed with a 5 mm thick quartz glass as a backing (e_b_ = 1500 Ws^1/2^m^−2^K^−1^, quartz glass is transparent in visible and near infrared), with silicone grease as cf2 and air as cf1. The obtained results for the three fitting parameters are *α_s_* = 0.03 cm^2^s^−1^, *e_s_* = 7510 Ws^1/2^m^−2^K^−1^ and *L_f2_* = 10 µm. The values of the thermal parameters of Cr_2_O_3_ are in agreement with previously performed results [4,12].

**Figure 6:**
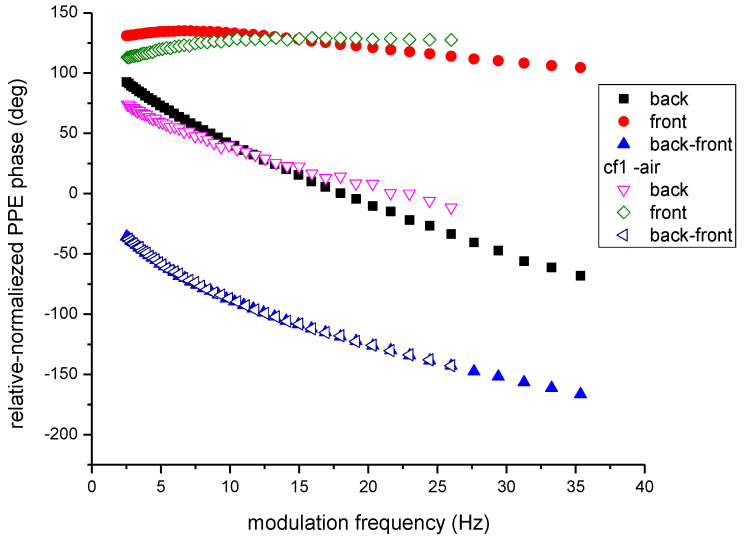
Behavior of the relative and normalized PPE phase as a function of the modulation frequency for Cr_2_O_3_ sample with silicone grease and air respectively as cf1.

**Figure 7:**
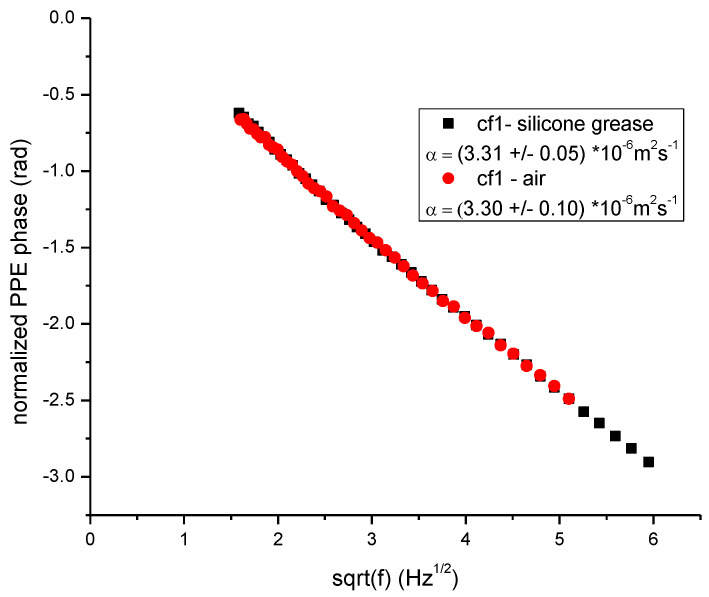
Behavior of the normalized PPE phase as a function of the square root of the modulation frequency for Cr_2_O_3_ sample in two configurations: cf1 air and silicone grease, respectively.

In order to compare the quality of the measurements performed with a good coupling fluid (silicone grease) and air respectively, as cf1, the same Cr_2_O_3_ single crystal was investigated. The backing material was PDMS and L_f2_ = 0. The results are displayed in Figure 6. As expected, the curves describing the phase behavior in the back and front configurations are different for the two runs (with different cf1). However, the normalized phases don’t depend on cf1, as predicted by the theory and pointed out in Figure 6. Air, as a coupling fluid between sensor and sample, acts as a low pass filter in the frequency scan and consequently, the frequency range is narrower than in the case when silicone grease was used as cf1. The signal to noise (S/N) ratio is also smaller when using air as cf1, but, however, good results can be obtained. Figure 7 displays the behavior of the normalized (back-front) PPE phase for Cr_2_O_3_ sample as a function of sqrt(*f*), in the two configurations: with cf1 air and silicone grease, respectively. The values obtained for the thermal diffusivity (from the slope of the curves in the high frequency range) for the two measurements are in good agreement and also in conformity with literature data [4,12,17]. Unfortunately, for this sample, the thermal effusivity cannot be obtained because the ratio e_s_/e_b_ > 10 and the method is not sensitive to the value of the sample’s thermal effusivity [12]. This example wants only to prove the quality of a measurement with air as cf1.

**Figure 8:**
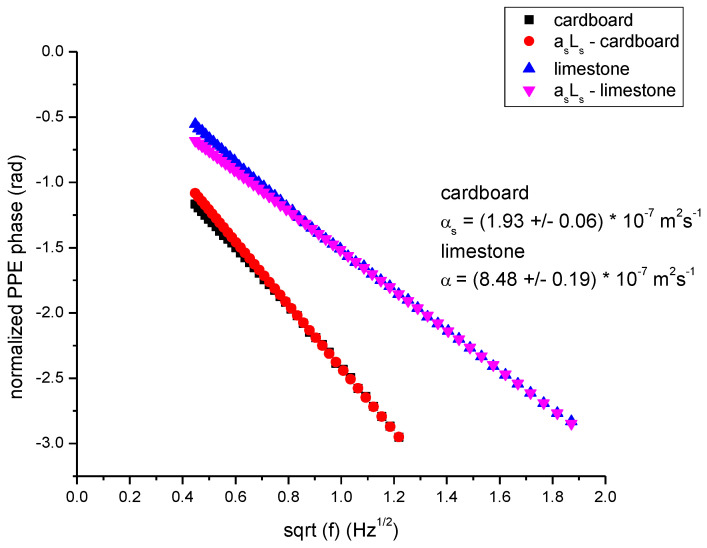
Behavior of the normalized PPE phase as a function of the square root of the modulation frequency for two porous samples: cardboard *L_s_* = 430 µm and limestone, *L_s_* = 810 µm, cf1 = air, backing PDMS, together with the linear contribution from high frequencies.

**Figure 9:**
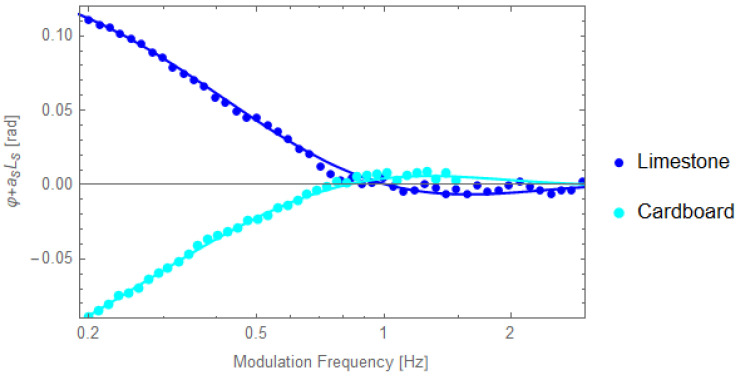
Behavior of *φ* + *a_s_L_s_* as a function of the modulation frequency for cardboard and limestone, together with the best fit performed with Equation (18). The fit used the value of the thermal diffusivity found from the slope of the curve at high frequencies.

Figure 8 and Figure 9 present some typical results obtained fortwo porous materials. Cardboard, a cellulose based material and limestone, a well-known building material, were selected as examples. Figure 8 presents the normalized PPE phase as a function of the square root of the modulation frequency, together with the behavior of the “linear” term (*a_s_L*_s_ vs sqrt(*f*)) in Equation (18). The slope of the *a_s_L_s_* (which is the same with that of the normalized PPE phase at high frequencies) gives the value of the thermal diffusivity.

Figure 9 presents the behavior of the *φ* + *a_s_L_s_* as a function of the modulation frequency together with the best fit performed by the use of Equation (18). In the fit, the value of the thermal diffusivity found from the slope of the curve at high frequencies was used. The results are in accord with literature data [20].

**Figure 10:**
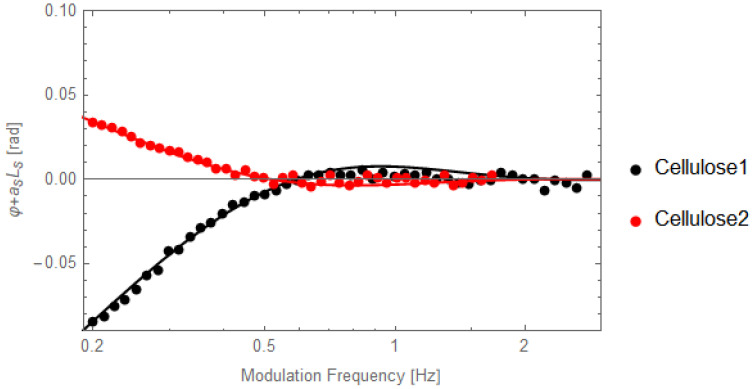
Behavior of *φ* + *a_s_L_s_* as a function of the modulation frequency for the two samples of cellulose-chitosan, together with the best fit performed with Equation (18).

Figure 10 presents the behavior of the *φ* + *a_s_L_s_* as a function of the modulation frequency for the two cellulose-chitosan samples, with different porosity, together with the best fit performed with Equation (18). As expected, the porosity influences drastically especially the thermal effusivity, due to the fact that air has a low thermal effusivity (5.5 Ws^1/2^m^−2^K^−1^).

**Figure 11:**
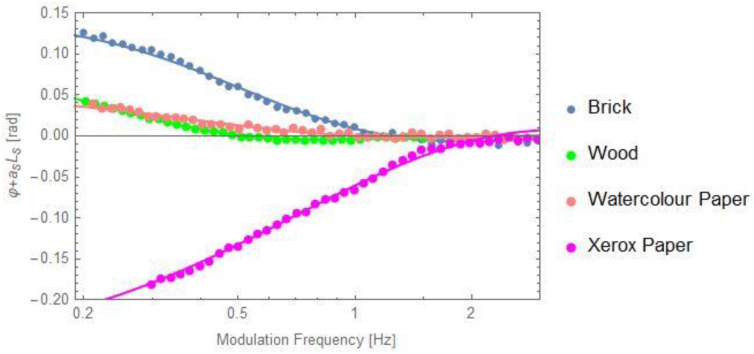
Behavior of φ + a_s_L_s_ as a function of the modulation frequency for other porous samples, together with the best fit performed with Equation (18).

Figure 11 displays the behavior of the *φ* + *a_s_L_s_* as a function of the modulation frequency for other porous samples: two building materials (brick and wood) and two types of paper (watercolour and xerox) together with the best fit performed with Equation (18). The results are also in good agreement with previously reported data [10,11,20].

Table 1 lists the thermal parameters of the investigated samples, as obtained from the measurements and as obtained by calculations. The uncertainties of the measured thermal parameters have been calculated as RMS of the best fit performed with Equation (18).

## 5. Conclusions

In this paper, we propose a PPE detection configuration based on an opaque sample and transparent pyroelectric sensor, backing and coupling fluids. In fact the theory developed here is an extension of the theory described in Ref. [12]. Compared with the previously reported configuration [12], it is upgraded with an additional transparent coupling fluid layer between the sample and sensor. In this configuration, a combined back-front detection investigation, based on a frequency scan of the phase of the PPE signals, followed by a self normalization of the phases behavior, leads to the possibility of measuring simultaneously both thermal effusivity and diffusivity of a solid sample. The result is obtained through a multiparametric fitting procedure with three fitting parameters, among which, two belong to the sample (thermal diffusivity and effusivity) and one to the experiment (thickness of the coupling fluid between sample and backing). However, more useful are the particular detection cases, because they can be applied to porous solids. One of them with air as a coupling fluid at both sample/sensor and sample/backing interfaces, leads to the possibility of measuring sample’s thermal diffusivity. Another particular case of this configuration, with no coupling fluid at the sample/backing interface and air instead of coupling fluid at the sample/sensor interface (non-contact method) is suitable for simultaneous measurement of both thermal diffusivity and effusivity (in fact complete thermal characterization) of porous solids. Compared with the already proposed configurations for investigations of porous materials [9,10,11], this one makes use of a fitting procedure with only one fitting parameter, in order to guarantee the uniqueness of the solution. The thermal diffusivity is obtained at high frequencies and thermal effusivity in the low frequency range.

From experimental point of view, the main novelty of the configuration proposed in the paper, is the insertion of air as a coupling fluid between the sensor and sample. Air, as a coupling fluid acts as a low-pass filter, and especially in the back detection configuration, it reduces the frequency range of investigations. However, we demonstrated in the paper that, even in this case, the measurements can be performed in an acceptable frequency range to obtain independently both thermal diffusivity and effusivity. Another particularity of this configuration is the backing material. Zammit et al. [12] used the same configuration, but for the thermal characterization of non-porous materials. Consequently, they could use high thermal quality coupling fluid between sensor and sample and, as backing, they used a liquid, when investigating solid samples. This is unlikely when investigating porous compounds. In such a case, the backing material must stick to the porous sample without penetrating the pores. Additional requirements for the backing material are: it must be transparent in both visible and infrared spectral ranges and its thermal effusivity must be in the range of 0.1–10 with the sample’s thermal effusivity. Out of this range, as demonstrated in [12], the method is not sensitive to the sample’s thermal effusivity. It seems that the backing material must face a lot of requirements; however, several gels fulfill these conditions. In the paper, we recommended two of them. We want finally to point out that the main request of this configuration is the opacity of the sample. If the sample is not opaque (too porous or too thin) only the methods proposed in Refs. [9,10,11] can be used.

Applications on several porous building materials and cellulose based samples validate the theory. When a comparison is made, the obtained results were in agreement with literature data.

As a final remark, the porous solids belong to a class of materials which are by far not easy to be investigated using PPE. To the best of our knowledge, the PPE technique was applied for thermal characterization of all types of condensed matter samples [21,22,23,24,25,26], the porous materials representing the only type of compounds, which were not taken into consideration (until recently). Consequently, the method proposed in this paper together with the alternative methods reported in Refs. [9,10,11], complete the area of applications of the PPE method.

## Figures and Tables

**Table 1:** Thermal parameters of the investigated samples, as obtained from the measurements (thermal diffusivity and effusivity) and as obtained by calculations (thermal conductivity and volume specific heat).

Sample	Thickness (µm)	Th. Diffusivity (10^7^ m^2^s^−1^)	Th. Effusivity (Ws^1/2^m^−2^K^−1^)	Th. Conductivity (10 Wm^−1^K^−1^)	Vol. sp. Heat (10^−6^ Jm^−3^K^−1^)
brick	700	7.72 ± 0.12	1084 ± 32	9.49 ± 0.05	1.23 ± 0.09
limestone	810	8.48 ± 0.19	1086 ±33	11.77 ± 0.06	1.38 ± 0.08
Wood (fir)	830	4.65 ± 0.09	744 ± 21	5.07± 0.04	1.09 ± 0.06
watercolour paper	300	1.15 ± 0.04	529 ±16	1.78 ± 0.04	1.56 ± 0.12
xerox paper	200	1.16 ± 0.04	106 ± 5	0.36 ± 0.02	0.31 ± 0.03
cardboard	430	1.93 ± 0.05	162 ± 6	0.71 ± 0.02	0.36 ± 0.03
cellulose (1)	630	2.79 ± 0.09	112 ± 5	0.58 ± 0.02	0.21 ± 0.02
cellulose (2)	600	2.37 ± 0.08	664 ± 18	3.23± 0.04	1.36 ± 0.08

## Data Availability

No new data were created.

## Supplementary Materials

The following supporting information can be downloaded at: https://www.mdpi.com/article/10.3390/ma16155242/s1.
