# Supplementary material for: Non-Contact and Self-Calibrated Photopyroelectric Method for Complete Thermal Characterization of Porous Materials
Source: Materials (Basel). 2023 Jul 26;16(15):5242. doi: 10.3390/ma16155242 (PMC10419518; doi:10.3390/ma16155242)
Supplement: Supplementary file 1 [file materials-16-05242-s001.zip › materials-2521061-supplementary.pdf]

Auxiliar material describing the system of differential equations with the corresponding initial and boundary conditions for the detection geometry considered in this paper.

For the geometry in Fig.1, the system of thermal diffusion equations can be written as:

$$\frac{d^2T_i}{dx_i^2} - \frac{i\omega}{\alpha_i}T_i = 0; \quad i = air,\ p,\ c1,\ s,\ c2,\ b$$

The solutions for the standard one directional heat diffusion system of equations are:

$$T_{air}(x) = Ae^{-\sigma_a(x-L_{f1}-L_p)}$$

$$T_p(x) = Qe^{\sigma_p(x-L_{f1})} + Pe^{-\sigma_p(x-L_{f1})}$$

$$T_{c1}(x) = Ue^{\sigma_{f1}x} + Ve^{-\sigma_{f1}x}$$

$$T_s(x) = Ce^{\sigma_s x} + Be^{-\sigma_s x}$$

$$T_{c2}(x) = Me^{\sigma_{f2}(x+L_s)} + Ne^{-\sigma_{f2}(x+L_s)}$$

$$T_b(x) = De^{\sigma_b(x+L_s+L_{f2})}$$

Boundary conditions of temperature and flux continuity:

-Front configuration

- Interface $L_{f1} + L_p$

$$T_{air}(x)/_{x=L_{f1}+L_p} = T_p(x)/_{x=L_{f1}+L_p}$$

$$A = Qe^{\sigma_p L_p} + Pe^{-\sigma_p L_p}$$

$$-k_a\frac{dT_{aer}(x)}{dx}/_{x=L_{f1}+L_p} + k_p\frac{dT_p(x)}{dx}/_{x=L_{f1}+L_p} = 0$$

$$k_p\sigma_p(Qe^{\sigma_p L_p} - Pe^{-\sigma_p L_p}) + k_a\sigma_a A = 0$$

- Interface L_{f1}

$$T_p(x)/_{x=L_{f1}} = T_{c1}(x)/_{x=L_{f1}}$$

$$Q + P = Ue^{\sigma_{f1}L_{f1}} + Ve^{-\sigma_{f1}L_{f1}}$$

$$k_{f1}\frac{dT_{c1}(x)}{dx}/_{x=L_{f1}} - k_p\frac{dT_p(x)}{dx}/_{x=L_{f1}} = 0$$

$$k_{f1}\sigma_{f1}(Ue^{\sigma_L L_{f1}} - Ve^{-\sigma_L L_{f1}}) - k_p\sigma_p(Q - P) = 0$$

- Interface x=0

$$\mathrm{T_{c1}(x)}/_{\mathrm{x=0}} = \mathrm{T_s(x)}/_{\mathrm{x=0}}$$

$$C + B = U + V$$

$$k_s \frac{dT_s(x)}{dx}/_{x=0} - k_{f1}\frac{dT_{c1}(x)}{dx}/_{x=0} = 0$$

$$k_s\sigma_s(-B + C) - k_{f1}\sigma_{f1}(U - V) = H$$

- Interface -L_s

$$T_s(x)/_{x=-L_s} = T_{c2}(x)/_{x=-L_s}$$

$$Be^{\sigma_s L_s} + Ce^{-\sigma_s L_s} = M + N$$

$$k_{f2}\frac{dT_{c2}(x)}{dx}/_{x=-L_s} - k_s\frac{dT_s(x)}{dx}/_{x=-L_s} = 0$$

$$k_{f2}\sigma_{f2}(M - N) - k_s\sigma_s(Ce^{-\sigma_s L_s} - Be^{\sigma_s L_s}) = 0$$

- Interface -L_s - L_{f2}

$$T_{c2}(x)/_{x=-L_s-L_{f2}} = T_b(x)/_{x=-L_s-L_{f2}}$$

$$Me^{-\sigma_{f2}L_{f2}} + Ne^{\sigma_{f2}L_{f2}} = D$$

$$k_b\frac{dT_b(x)}{dx}/_{x=-L_s-L_{f2}} - k_L\frac{dT_{c2}(x)}{dx}/_{x=-L_s-L_{f2}} = 0$$

$$k_b\sigma_b D - k_{f2}\sigma_{f2}(Me^{-\sigma_{f2}L_{f2}} - Ne^{\sigma_{f2}L_{f2}}) = 0$$

- **Back configuration**

The boundary conditions are the same as in the front configuration for the interfaces:L_{f1}+L_p, L_{f1}, -L_s-L_{f2}

Interface 0:

$$C + B = U + V$$

$$k_s\sigma_s(-B + C) - k_L\sigma_L(U - V) = 0$$

Interface -L_S:

$$Be^{\sigma_s L_s} + Ce^{-\sigma_s L_s} = M + N$$

$$k_L \sigma_L (M - N) - k_s \sigma_s (Ce^{-\sigma_s L_s} - Be^{\sigma_s L_s}) = H$$

Using the system of heat diffusion equations with the boundary conditions described above we obtained the results presented in the paper.
